# Behaviour of Titanium Dioxide Particles in Artificial Body Fluids and Human Blood Plasma

**DOI:** 10.3390/ijms221910614

**Published:** 2021-09-30

**Authors:** Eva Korábková, Věra Kašpárková, Daniela Jasenská, Dita Moricová, Eliška Daďová, Thanh Huong Truong, Zdenka Capáková, Jan Vícha, Jana Pelková, Petr Humpolíček

**Affiliations:** 1 Centre of Polymer Systems, Tomas Bata University in Zlin, nám. T. G. Masaryka 5555, 76001 Zlin, Czech Republic; e_korabkova@utb.cz (E.K.); vkasparkova@utb.cz (V.K.); jasenska@utb.cz (D.J.); e_dadova@utb.cz (E.D.); truong@utb.cz (T.H.T.); capakova@utb.cz (Z.C.); jvicha@utb.cz (J.V.); 2 Faculty of Technology, Tomas Bata University in Zlín, nám. T. G. Masaryka 5555, 76001 Zlin, Czech Republic; d_moricova@utb.cz; 3 Department of Hematology, Tomas Bata Regional Hospital in Zlin, 76275 Zlin, Czech Republic; Jana.Pelkova@bnzlin.cz

**Keywords:** TiO_2_ particles, simulated gastric fluids, proteins, plasma, agglomeration

## Abstract

The growing application of materials containing TiO_2_ particles has led to an increased risk of human exposure, while a gap in knowledge about the possible adverse effects of TiO_2_ still exists. In this work, TiO_2_ particles of rutile, anatase, and their commercial mixture were exposed to various environments, including simulated gastric fluids and human blood plasma (both representing in vivo conditions), and media used in in vitro experiments. Simulated body fluids of different compositions, ionic strengths, and pH were used, and the impact of the absence or presence of chosen enzymes was investigated. The physicochemical properties and agglomeration of TiO_2_ in these media were determined. The time dependent agglomeration of TiO_2_ related to the type of TiO_2_, and mainly to the type and composition of the environment that was observed. The presence of enzymes either prevented or promoted TiO_2_ agglomeration. TiO_2_ was also observed to exhibit concentration-dependent cytotoxicity. This knowledge about TiO_2_ behavior in all the abovementioned environments is critical when TiO_2_ safety is considered, especially with respect to the significant impact of the presence of proteins and size-related cytotoxicity.

## 1. Introduction

Due to its favorable properties, such as good biocompatibility, outstanding stability, high refractive index, corrosion resistance, and low-cost production, titanium dioxide (TiO_2_) is recognized to be one of the most interesting nanomaterials. This nanomaterial is widely used as a white pigment in paints, sunscreens, cosmetics, toothpaste, and foods. Thanks to the abovementioned properties, the application of TiO_2_ has also substantially increased in sensors, catalysis, photocatalysis, and biomedicine [1,2,3]. Furthermore, the antibacterial effect of nano-sized TiO_2_ particles has been confirmed not only against the gram negative *Escherichia coli* strain but also against pathogenic species [4,5,6].

TiO_2_ exists in three main crystalline forms, rutile (tetragonal), anatase (tetragonal), and, more rarely, brookite (orthorhombic). Rutile is thermodynamically the most stable form, whereas anatase and brookite are metastable and are converted to rutile by heating [7,8]. The mentioned variations in crystalline forms influence the biological properties of TiO_2_ nanoparticles (NPs), which may interact with biological systems in different ways. For example, [9] described the greater cytotoxic, neoplastic, and genotoxic effects of rutile compared to anatase. The biological properties of TiO_2_ NPs are related not only to their form, but also to other physicochemical properties such as particle size, specific surface area, surface potential, etc. [10,11]. In this context, the wide use of TiO_2_ NPs requires careful consideration of the potential risks to human health as well as of the potential impacts on the environment.

TiO_2_ NPs can penetrate into the human body via several different pathways, including oral, dermal, and inhalation routes [2]. Due to the presence of TiO_2_ in many dermal and cosmetic formulations such as sunscreens, powders, eyeshadows, creams, foundations, and lip balms, the exposure of humans to TiO_2_ NPs via the oral route or through the skin is inevitable [11,12,13]. In 2016, the Commission Regulation (EU) 2016/1143 allowed the use of TiO_2_ in the form of NPs at concentrations of up to 25% as a UV filter, though not in spray products that may lead to exposure of the lungs through inhalation. In fact, human data concerning the effects of exposure to TiO_2_ by inhalation are limited. As regards topical applications, according to most studies carried out on humans or animals, no penetration of TiO_2_ NPs beyond the outer layers of the *stratum corneum* to viable *epidermis* or *dermis* cells has been proven after dermal exposure [14,15]. However, Pelclova et al. recently reported the potential absorption of a minimal amount of TiO_2_ NPs by the skin after the use of a commercial sunscreen due to its presence in plasma and urine of humans [16]. Despite the limitations of this experiment (there were only six participating volunteers), this is the first study to indicate that further evaluation of the safety of TiO_2_ NPs in topical products is necessary. Owing to the accidental oral intake of some types of cosmetic products such as lip balms, it is also necessary to consider the possibility of the penetration of TiO_2_ NPs into oral and gastrointestinal mucosa [13]. A human in vivo study on the gastrointestinal absorption of TiO_2_ NPs of different particle sizes was carried out by [17], who reported the absence of significant TiO_2_ NPs absorption after oral exposure in humans, independent of particle size [17]. In a study conducted by Cho et al., they examined the kinetics of TiO_2_ and ZnO NP absorption in vivo and demonstrated that TiO_2_ NPs were absorbed significantly less than ZnO NPs, which can be explained by the higher dissolution of ZnO compared with TiO_2_ NPs [2]. Recently, Marucco et al. conducted a study to evaluate the biotransformation of food-grade and nanometric TiO_2_ during the transit in the oral-gastro-intestinal tract using a simulated human digestive system. The results showed particle aggregation as a result of the high ionic strength in the gastric and intestinal simulated fluids. The authors further observed the formation of a hard-biocorona, resulting in partial masking of the TiO_2_ particles surface and reactivity [18]. In addition, Dudefoi et al. dealt with the fate of TiO_2_ particles in the gastrointestinal tract and their effect on the activity of digestive enzymes. The authors reported the formation of agglomerates mainly in the intestinal fluid due to the adsorption of α-amylase and divalent cations. Moreover, in saliva, TiO_2_ inhibited the enzymatic activity of α-amylase, whereas in gastric fluid, the activity of pepsin was not affected [19].

Generally, the penetration of TiO_2_ NPs via the gastrointestinal tract (GIT) into the blood circulation system depends on different factors. However, absorption from the GIT into blood and urine is rare, and recent studies suggest that after ingestion, most particles are discharged by the GIT [20,21]. According to [12], TiO_2_ nanoparticles could, however, accumulate in tissues and organs such as a the liver, spleen, kidneys, and lungs after absorption by the GIT. Thus, even limited penetration from the GIT, or skin, into blood could have an important impact if long-term exposure is considered. The effect of particle size on the toxicity and accumulation of TiO_2_ after a single dose of 5 g/kg body weight was demonstrated by [12] in a study on mice. Larger particles (80 nm) accumulated in the liver, while smaller particles (25 nm) were observed in the spleen, kidneys, and lungs. In this study, no evident acute toxicity of TiO_2_ was proven. Gender-dependent effects were, however, evident after exposure to TiO_2_ nanoparticles; specifically, liver and kidney damage was recorded in female mice. Nevertheless, the very high dose of TiO_2_ NPs used in this study is not typical for human exposure. The results of this study conform with the in vivo study in mice performed by [22], who found the highest accumulation of nano-sized TiO_2_ particles in the spleen. TiO_2_ NPs were also present in the liver, kidney, and lung. Several in vivo studies indicated that TiO_2_ NPs are capable of penetrating the intestinal mucosa after oral exposure [23]. All these studies indicate a potential risk of the accumulation TiO_2_ NPs in specific organs after repeated long-term oral exposure.

In the context of incidental oral exposure through oral use, we present the results of a study focused on comparing the in vitro behaviors of TiO_2_ particles in the form of rutile, anatase, and their mixture in simulated body fluids of the GIT (saliva and simulated gastric and intestinal fluids). As an understanding of TiO_2_ behavior in a biological environment is essential for in vitro studies, the fate of TiO_2_ particles in cell culture medium (DMEM) and phosphate buffered saline (PBS) is reported. The study is also enriched with a description of the agglomeration behavior of TiO_2_ by monitoring the direct contact of particles with blood plasma, which can occur when epithelium or skin are injured. The main novelty of the research relies not only in the unique comparison of TiO_2_ particles (rutile, anatase and mixture) exposed to simulated gastric fluids, human blood plasma, and media used in in vitro experiments, but also in the comparison of their cytotoxicity (ATP assay), including information on the number of healthy, necrotic, and apoptotic cells acquired by flow-cytometry. Another key contribution of the presented study is the following of the time-dependent agglomeration of TiO_2_ under comparable conditions. As identical methods and procedure were used, the reported behavior of the samples can facilitate comparison of various samples when conducting biological testing of TiO_2_ particles.

TiO_2_ is not always correctly referred to as nanoparticles/particles in the literature. In our text, however, we follow the legislation rules defining nanoparticles as materials with one or more external dimensions on the scale from 1 to 100 nm, and therefore, we designate our samples as TiO_2_ particles.

## 2. Materials and Methods

### 2.1. Titanium (IV) Oxide Particles

Titanium (IV) oxide particles with different sizes and crystallographic phases were used in the study (Table 1). Samples labelled as Rutile 1 (Cat No. 120717/1) and Rutile 5 (Cat No. 120717/5) were the kind gift of Precheza a. s. (Přerov, Czech Republic). A mixture of Rutile/Anatase (Cat No. 634662-25G) and Anatase (Cat No. 637254-50G) were purchased from Sigma Aldrich (Taufkirchen, Germany).

### 2.2. Simulated Body Fluids, Blood Plasma, and Cultivation Media

Artificial saliva solution (Saliva) was prepared by dissolving 0.03 g NaNO_2_, 0.2 g K_2_CO_3_, 0.5 g NaCl, and 4.2 g NaHCO_3_ in 1 L water (Milli-Q filtration system, Merck, Darmstadt, Germany). The solution was adjusted to pH 6.8 with 1 M NaOH or 1 M HCl (all from Sigma-Aldrich, Taufkirchen, Germany). Simulated gastric fluid (SGF) (pH 1.2) was prepared by dissolving 1 g NaCl in 40 mL 1 M hydrochloric acid. Milli-Q water was added to make a final volume of 0.5 L, and the solution was adjusted to pH 1.2 with 1 M HCl. Similarly, simulated gastric fluid with pepsin (SGF^Pepsin^) with pH 1.2 was prepared by the addition of 1.6 g pepsin (pKa = 1.57; 5.02 [24], Sigma Aldrich, (Taufkirchen, Germany) to 0.5 L of the SGF. Simulated intestinal fluid (SIF) with pH 6.8 was prepared by dissolving 6.8 g KH_2_PO_4_ in 77 mL 0.2 M NaOH and adding water to make a final volume of 1 L. Then, the solution was adjusted to pH 6.8 with 1 M NaOH or 1 M HCl. For SIF with pancreatin (SIF^Pancreatin^) with pH 6.8, pancreatin (pKa = 5 [25], Sigma Aldrich, (Taufkirchen, Germany) was further added to the solution at a concentration of 10 g/L [26]. Human blood plasma was obtained from healthy individuals via the Tomas Bata Regional Hospital in Zlín, after informed consent was signed. In addition to the described simulated body fluids, Phosphate buffered saline (PBS), Dulbecco’s Modified Eagle Medium (DMEM), and DMEM supplemented with 10% Calf serum (DMEM^Serum^), commonly used for cultivation of fibroblast cells [27], were used to reveal the behavior of TiO_2_ NPs (all purchased from BioSera, Nuaille, France).

### 2.3. Preparation of TiO_2_ Dispersions

TiO_2_ dispersions were prepared by homogenizing 0.05 g TiO_2_ particles in 10 mL Milli-Q water for 30 min using a UP400S sonicator (Heielscher, Teltow, Germany) at a power of 400 W (24 kHz) and using an amplitude of 100% with a cycle setting of 0.6 on an ice bath. The dispersions were then mixed with the fluids defined above in concentrations specified below.

### 2.4. Characterization

#### 2.4.1. Dynamic Light Scattering

The particle sizes and particle size distributions of TiO_2_ particles were determined by dynamic light scattering (DLS) on a Zetasizer Nano ZS90 (Malvern Instruments, Malvern, Worcestershire, UK). Analyses were performed after dilution of the initial TiO_2_ dispersions in the given media at a scattering angle of 90° and temperature of 25 °C. For measurements, 20 µL of dispersion was added to 1 mL of each medium. The time-dependent development of particle size was studied for 100 min at 20 min intervals. Particle size was expressed as an average intensity-derived particle diameter (z-average diameter) calculated on the basis of at least three repeated measurements. In addition to particle size, the polydispersity index (PDI) was also obtained. The PDI is a measure of the width of the distribution and takes values from 0 to 1, where values ~0.1 would be achieved by a completely monodisperse system and values greater than 0.7 denote a sample with a very broad size distribution.

#### 2.4.2. Zeta Potential

The Zeta potential was measured using a Zetasizer Nano ZS90 (Malvern Instruments, Worcestershire, Malvern, UK). Measurements were performed in Milli-Q water with pH varying within the pH range of 1 to 10. For measurements, 15 µL TiO_2_ dispersion was mixed with 4 mL pH adjusted water to a final concentration of 3.75 µL/mL. Zeta potential measurements are expressed as means and standard deviations and are calculated on the basis of at least three repeated measurements.

#### 2.4.3. Scanning Electron Microscopy (SEM)

The morphology of TiO_2_ particles was determined using a Nova NanoSEM 450 scanning electron microscope (SEM) (FEI, ThermoFischer Scientific, Waltham, MA, USA) at an accelerating voltage of 5 kV.

#### 2.4.4. Cytotoxicity

In order to investigate the cytotoxic effect of TiO_2_ particles, mouse embryonic fibroblast cell lines (ATCC CRL-1658 NIH/3T3) were used. ATCC–formulated Dulbecco’s Modified Eagle’s Medium (DMEM, PAA Laboratories GmbH, Pasching, Austria) containing 10% calf serum (Biosera, Nuaille, France) and 1% Penicillin/Streptomycin (GE Healthcare HyClone, Northumberland, UK) was used as the culture medium. Cytotoxicity evaluation was performed according to ISO protocol 10 993-5:2009. Dispersions of TiO_2_ were prepared by diluting the 5.0 mg/mL parent dispersion, this step was followed by homogenization in 10 mL culture medium for 30 min using a UP400S sonicator (Heielscher, Teltow, Germany). A series of concentrations—5.0, 4.0, 3.5, 3.0, 2.5, 2.0, 1.5, 1.0 mg/mL—was obtained. For the cytotoxicity assay, NIH/3T3 cells were pre-cultivated for 24 h and seeded at a concentration of 1 × 10^5^ per well (96 well plates were used, TPP Trasadingen, Switzerland). The culture medium was subsequently replaced by TiO_2_ dispersions with the above-given concentrations. The cytotoxicity was measured 24 h after exposure. As a reference, cells cultivated in pure medium without TiO_2_ particles were used. Cytotoxic effects were assessed using the ATP Determination Kit (A22066, Thermo Fisher Scientific, Waltham, MA, USA). The luminescence was measured with an Infinite M200 PRO luminometer (Tecan, Männedorf, Switzerland). All tests were carried out in quadruplicates in two independent tests. Results are presented as the relative values of cell viability compared to cells cultivated in medium without the presence of the tested materials (reference with viability 1). Cell morphology was observed using an inverted Olympus phase contrast microscope (Olympus IX81, Tokyo, Japan).

## 3. Results and Discussion

Rutile and anatase are the most commonly applied crystalline forms of TiO_2_. Though they have the same chemical composition, their structures are different [28,29], with this conditioning their behavior, including interactions with physiological media and body fluids.

### 3.1. Morphology of TiO_2_ Particles

**Figure 1:**
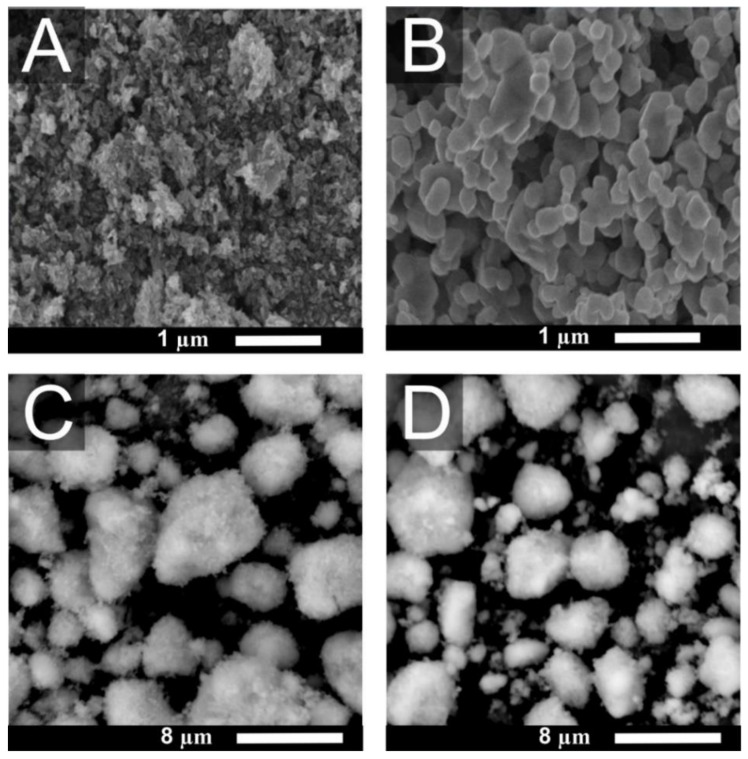
SEM micrographs of (**A**) Rutile 1, (**B**) Rutile 5, (**C**) Anatase, and (**D**) Anatase/Rutile.

TiO_2_ particles were visualized by SEM. The Figure 1 clearly illustrates differences in shape and morphology among the individual forms of the particles. While Rutile 1 (Figure 1A) appears as clusters of elongated needle-like particles, Rutile 5 contains spherical particles with a clear crystal contour and a smooth surface (Figure 1B). The figures also show differences in the size of primary crystallites—specifically, 21 nm and 150 nm for Rutile 1 and Rutile 5, respectively. Commercial Anatase and Anatase/Rutile samples displayed similar morphology. The particles are spherical with an irregular shape and a rough surface (Figure 1C,D). The morphology corresponds to the study of [30].

### 3.2. Behaviour of TiO_2_ Particles in Water

Before mixing TiO_2_ particles with physiological media and simulated fluids, their particle size and polydispersity index (PDI) were determined in Milli-Q water to acquire reference values. The results from DLS listed in Table 2 show the z-average diameters of particle sizes ranging from 142 ± 2 nm to 553 ± 7 nm, and PDI values ranging from 0.18 ± 0.01 (Rutile 5) to 0.35 ± 0.01 (Anatase/Rutile). This points to the presence of particles with different sizes and distributions.

**Figure 2:**
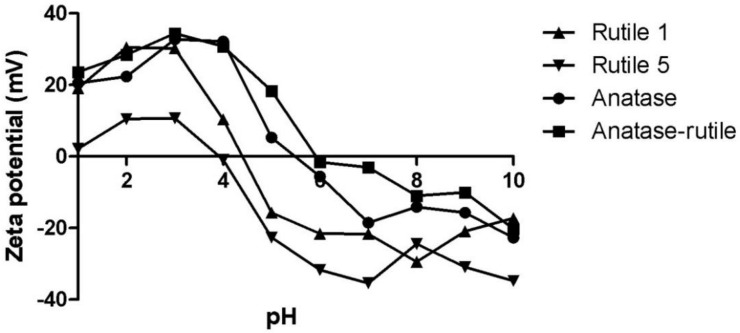
Development of zeta potential of TiO_2_ particles with the pH.

Zeta potentials were followed at various pH (Figure 2) and the course of ζ vs. pH dependencies exhibited a similar trend. The isoelectric point (IEP), however, varied within the pH range of ~4–6, and both Rutile samples displayed IEPs below pH 5, in contrast to Anatase and Anatase/Rutile samples, which exhibited IEPs of 5.5 and 6.0, respectively. Most commercial TiO_2_ nano-powders exhibit IEPs ranging widely from pH 2–9 [31]. The differences can be attributed to various factors, such as the insufficient surface purity of TiO_2_ NPs [10] or the size and shape of NPs [32]. Low IEP values resulting from the presence of phosphates or other anionic impurities in commercial TiO_2_ was observed by [33]. In this context, a study by Suttiponparnit et al. suggested that the crystallographic structure of TiO_2_ NPs has no significant effect on IEP [32].

### 3.3. TiO_2_ Particles in Cultivation Media

A battery of in vitro biocompatibility tests is routinely conducted in PBS and DMEM with or without serum. These media are close to the environment particles that meet in vivo in tissues. Following determination of the behavior of TiO_2_ particles in water, the in vitro fate of TiO_2_ particles in media was investigated immediately after the mixing of particles with each medium and at various time points after sample preparation. These data are noteworthy if experimental results from biocompatibility testing are evaluated.

**Figure 3:**
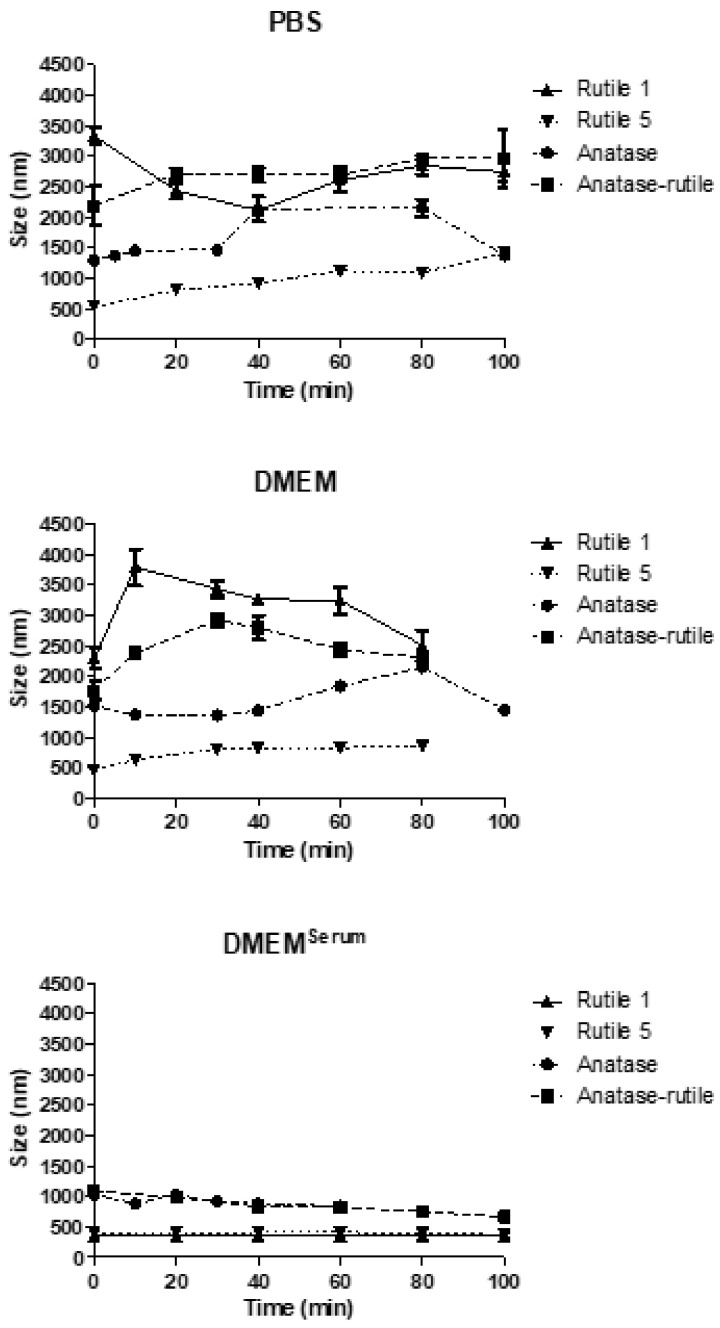
The time-dependent change in particle size of TiO_2_ particles in media used for in vitro experiments. The starting concentration of the TiO_2_ particles was 0.5 mg/mL dispersion media.

As expected, immediately after the mixing of the TiO_2_ dispersion with PBS, the sizes of the particles increased, which was most significant for Rutile 1 (from 215 ± 1 nm to 3330 ± 190 nm), thus demonstrating rapid agglomeration in the samples (Figure 3). Over time, the size of particles/agglomerates varied, but still remaining within the range of 2–3 μm. Similar behavior was observed with Anatase/Rutile and Anatase, but with different initial agglomeration levels. In addition, the size of Anatase particles decreased after 80 min of contact with PBS, which could be due to a size-triggered sedimentation of the formed agglomerates. In contrast, Rutile 5 particles agglomerated least and their size grew gradually during the experiment. The observed ionic strength-triggered increase in size is consistent with the study by Teubl et al., who observed a high level of TiO_2_ NPs agglomeration in PBS due to the presence of ions [34]. Moreover, Sager et al. pointed out the unsuitability of using PBS as a medium for preparing suspensions of nano-sized particles due to the formation of aggregates [35].

In serum free DMEM, samples also agglomerated thanks to the presence of electrolytes screening the particle surface charge. After the initial increase in particle size observed immediately after dilution with DMEM, the behavior of individual samples differed (Figure 3). However, the extent of agglomeration in DMEM was roughly similar as in PBS, with Rutile 5 particles showing both the smallest initial agglomeration and a lower agglomeration rate over time.

Additionally, here, the behavior of particles was strongly influenced by the ionic strength of the medium used for their dispersion. This is consistent with the study by Allouni et al., who studied the colloidal stability of TiO_2_ NPs in cell culture medium and observed growing agglomeration at increased TiO_2_ NPs concentrations due to the increasing frequency of direct particle-to-particle interactions resulting from the ionic strength of the medium [10].

The behavior of TiO_2_ particles, however, changed after the addition of 10% calf serum to DMEM (Figure 3), this leading to notably lower agglomeration in all dispersions. Rutile 1 and 5 increased their sizes the least and behaved almost the same during the entire measurement period. The Rutile/Anatase mixture and Anatase then agglomerated somewhat more; however, the size increase was far from achieving the sizes recorded in samples mixed with DMEM without serum. Here, it is obvious that calf serum hindered TiO_2_ particle interactions through the formation of a protective protein layer, a corona, on the surface of the particles. The serum, hence, exerts a stabilizing effect leading to the prevention of TiO_2_ particles agglomeration. Moreover, Ji et al., who studied the effect of calf serum on the size and stability of TiO_2_ NPs in different culture media, concluded that even 1% serum effectively stabilized dispersions and prevented NPs from agglomerating thanks to the content of specific proteins forming a protein corona [36]. The influence of fetal bovine serum (FBS), a common supplement for cell culture, and human serum albumin (HSA), the main component of blood plasma, on the colloidal stability of TiO_2_ NPs in cell culture medium was investigated in the work of [10]. The authors reported that the agglomerate size was considerably reduced upon the addition of 10% (*v*/*v*) FBS and 1% (*w*/*w*) HSA, and that both proteins thus prevented agglomeration and yielded a stable TiO_2_ NP dispersion. Biological fluids generally include various types of molecules such as proteins, enzymes, lipids, etc., that compete in adsorption to the NP surface. However, in most cases, these are the proteins that are primarily involved in corona formation as a result of protein–nanoparticle interactions [37]. The layer of adsorbed proteins on the surfaces of NPs gives them a new identity due to the change in their physicochemical properties, such as size, surface charge, surface composition, and functionality [38]. The protein corona (PC) consists of soft and hard layers, of which the former contains proteins with high affinity and direct interaction with the NP surface and the latter includes low-affinity proteins. The soft corona proteins then interact with the hard corona via weak protein-protein interactions [37,39,40]. The formation of the corona depends mainly on the physicochemical properties of NPs, the composition of the biological medium, and the interaction time [39,41].

According to the comparison of TiO_2_ particle behavior in the media examined above (Supplementary Figure S1a–d), the smallest agglomeration in all media was observed in Rutile 5. Immediately after mixing Rutile 5 with the fluid, the particle size increased from 287 ± 8 nm (water) to ~500 nm; then, as the measurement time increased, the particles gradually agglomerated—the most in PBS and to a lesser degree in DMEM. In DMEM^Serum^, they remained constant in size. In contrast to Rutile 1, the behavior of Anatase and Anatase/Rutile did not differ much in PBS and DMEM without serum, which can be attributed to the markedly different structure of Rutile 1, as confirmed by the SEM images.

The simulated gastrointestinal fluids were used to describe TiO_2_ particles with respect to their agglomeration after ingestion. Though the authors are aware of the simplicity of the used in vitro approach relative to the real situation, such tests can, however, contribute to an understanding of the performance of different TiO_2_ particles forms in vivo.

#### 3.3.1. Simulated Saliva

**Figure 4:**
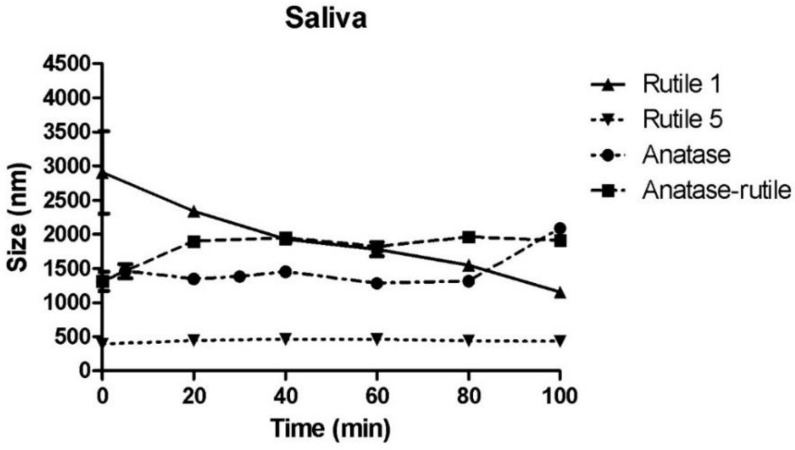
Time-dependent change in particle size of TiO_2_ particles in simulated saliva. The starting concentration of the TiO_2_ particles was 0.5 mg/mL dispersion media.

Saliva present in the oral cavity represents the first component of the gastrointestinal system, which rapidly interacts with NPs [34]. Correspondingly to the TiO_2_ particles dispersed in physiological media discussed above, the particles in simulated saliva (pH 6.8) agglomerated (Figure 4). For Rutile 1, the maximum size of agglomerates/particles was recorded immediately after mixing with saliva (2908 ± 854 nm); these agglomerates/particles then gradually decreasing in size during the prolonged time of mutual contact with the saliva. Rutile 5 particles changed the least due to the action of saliva. After an initial increase from 287 ± 8 nm (water) to 392 ± 13 nm (saliva), the particle diameter remained almost unchanged for the remainder of the measurement time. In Anatase/Rutile and Anatase, significant agglomeration was observed, though it was minor in comparison with Rutile 1. The results presented here are consistent with a study by Teubl et al., which reported a significant agglomeration of TiO_2_ NPs in simulated saliva independent of the surface properties (hydrophilicity/hydrophobicity) of the NPs [34]. Additionally, Sohal et al. investigated anatase NPs in simulated saliva by evaluating statistically significant changes in NP concentration over a range of nanoparticle sizes, and analyzed the anatase particles at two time points: immediately after mixing and then 60 min after mixing. The authors did not observe any notable changes in the concentration of anatase nanoparticles in the simulated saliva across the range of the investigated nanoparticle sizes [42]. The results of our study comply with the observations presented in the Sohal’s paper, showing a virtual steady state with respect to the size of TiO_2_ NPs during the entire measurement time. However, our results also show that agglomerates on particles form rapidly and immediately after mixing with saliva. On the other hand, these agglomerates may disintegrate and equilibrium may be established between agglomerated and non-agglomerated particles.

#### 3.3.2. Simulated Gastric Fluid

**Figure 5:**
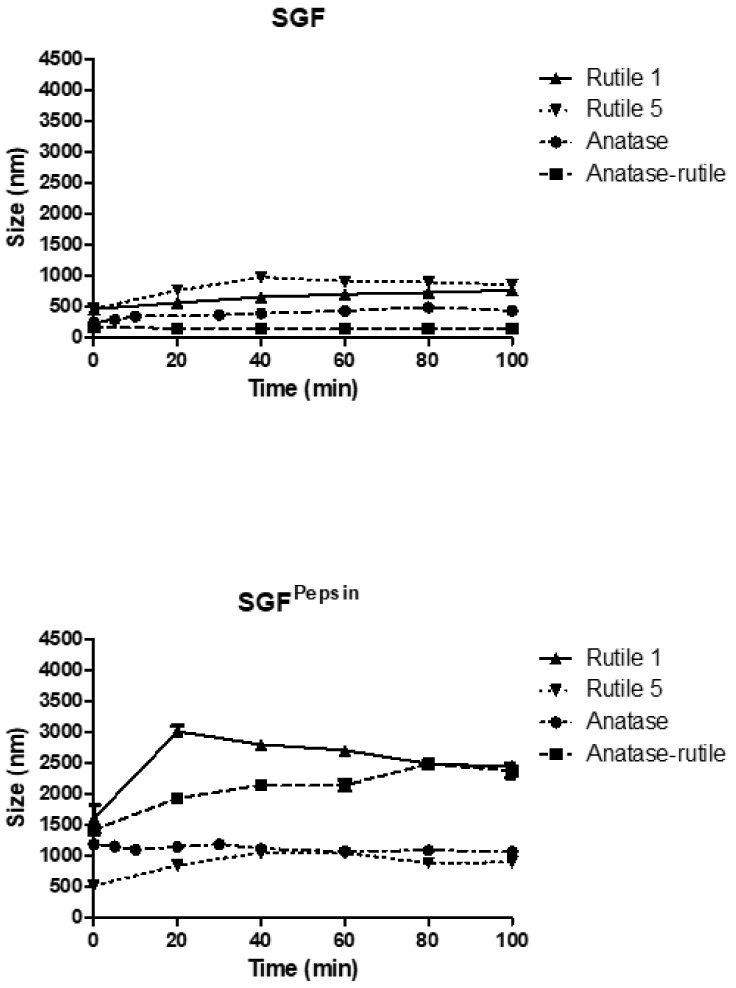
Time-dependent change in particle size of TiO_2_ particles in simulated gastric fluids. The starting concentration of the TiO_2_ particles was 0.5 mg/mL dispersion media.

After swallowing, the particles pass through the esophagus into the stomach and come into contact with gastric fluid. When mixed with SGF (pH 1.2), the behavior of the TiO_2_ particles was fundamentally different from that observed in other simulated body fluids (Figure 5). In SGF, particles of Rutile 1 increased their size to 459 ± 16 nm and, due to the slow aggregation, their size grew further to a final value of 762 ± 21 nm. Overall, the particles grew three times in size from the original value of 215 ± 1 nm determined in Milli-Q water. Similarly, the size of Rutile 5 particles increased, reaching a slightly higher maximum size of 973 ± 24 nm. Anatase/Rutile particles were the most stable in the SGF environment, with their size increasing only to a maximum value of 169 ± 10 nm from a reference value of 142 ± 2 nm in Milli-Q and changing only slightly with the measurement time. Anatase in SGF revealed a lower particle size than in water, reaching, after mixing, a size of 241 ± 9 nm, which then increased steadily to a maximum value of 478 ± 17 nm during the measurement time. Thus, the low pH of SGF (1.2) appears to contribute to lower particle agglomeration compared to other simulated fluids, though the charge and agglomeration of TiO_2_ NPs also strongly depends on the pH [43]. In this context, Cho et al. reported on TiO_2_ NPs incubated in acidic gastric fluid (pH 1.5–2.0) and water (pH 7.4), with their results showing only a minimal dissolution of NPs in both acidic gastric fluid and water [2].

In SGF^Pepsin^, the particles of all samples agglomerated extensively despite the low pH of the fluid (Figure 5). In Rutile 1, a particle size of 1605 ± 320 nm was measured immediately after mixing with SGF, which grew sharply to 3014 ± 124 nm, and then gradually decreased to a final size of 2444 ± 76 nm after 100 min. The particles of Rutile 5 showed an initial size of 512 ± 52 nm, which increased to around 1000 nm during the measurement. Anatase/Rutile particles grew from an initial size of 1417 ± 313 nm to a maximum value of 2485 ± 95 nm, and the particles of Anatase grew immediately after mixing with the fluid to a value of 1189 ± 134 nm, which did not change further during the test. The fate of TiO_2_ NPs in SGF supplemented with pepsin was discussed by [44], who reported on the formation of a soft corona with low affinity between TiO_2_ NPs and pepsin; correspondingly to our study, corona formation on the TiO_2_ NPs surface did not reduce NP aggregation [44]. An explanation can probably be found in the different charges of TiO_2_ NPs and pepsin; specifically, at acidic pH, TiO_2_ NPs in the presence of SGF without pepsin showed a positive charge, whereas pepsin in SGF displayed a minor negative charge. Therefore, protein corona formed due to relatively weak electrostatic interactions between NP and pepsin might not sufficiently separate NPs, thus allowing their agglomeration. The surface charge plays an important role in protein binding to NPs [45] and protein adsorption at the NP interface can lead to a change in the function of the proteins by inhibiting enzyme activity or causing structural changes in them [44,46]. These authors recorded a decrease in pepsin activity; however, in their work, no significant effect on pepsin secondary structure was noted in the presence of TiO_2_ NPs. On the basis of these presented results, it can be hypothesized that NPs may inhibit pepsin activity in the human stomach. In addition, McCracken et al. reported on the formation of a protein corona composed of bile acids and proteins after the incubation of TiO_2_ NPs in simulated gastrointestinal fluid [47].

#### 3.3.3. Simulated Intestinal Fluid

**Figure 6:**
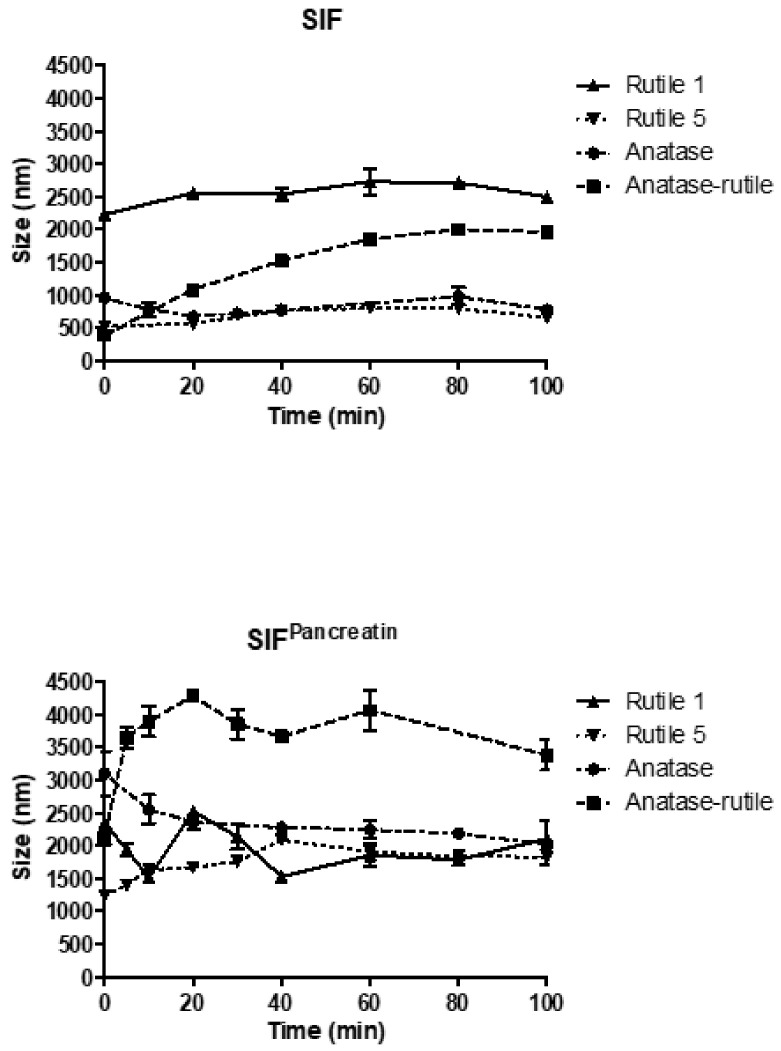
Time-dependent change in particle size of TiO_2_ particles in simulated intestinal fluid. The starting concentration of the TiO_2_ particles was 0.5 mg/mL dispersion media.

The agglomeration of TiO_2_ particles was also recorded in simulated intestinal fluid of pH 6.8 (Figure 6). Rutile 1 changed its particle size most significantly, correspondingly to the situation in simulated saliva. At the start of measurement, a size of 2226 ± 79 nm was detected, which remained almost stable during analysis. In contrast, Rutile 5 showed considerably less agglomeration, with an initial increase in particle size to 520 ± 58 nm, followed by further growth to a maximum value of 797 ± 79 nm, and then a decrease to 650 ± 27 nm at the end of the measurement, indicating agglomerate sedimentation. In Anatase/Rutile, a progressive agglomeration of particles was observed, reaching a maximum size of 1998 ± 40 nm. For Anatase, the particle sizes fluctuated during the analysis, with values below 1000 nm.

The time-dependent development of particle size in SIF^Pancreatin^ shown in Figure 6 demonstrates that pancreatin triggered significant TiO_2_ particle agglomeration (similarly to pepsin in SGF). The greatest changes were recorded for Anatase/Rutile, in which the particle/agglomerate size increased dramatically up to a maximum value of 4286 ± 41 nm measured after 20 min, and then decreased slightly, indicating the sedimentation of agglomerated particles. Similar to Anatase, significant agglomeration also occurred in both Rutile samples, of which Rutile 5 formed agglomerates less dramatically. A steady increase in the concentration of TiO_2_ (Anatase) nanoparticles in simulated intestinal fluid together with a notable increase in agglomerate size over time was also reported by Sohal et al. [42]. As regards ion release from TiO_2_ NPs in SIF, this study again confirmed the biodurable and persistent nature of TiO_2_ NPs.

Comparison of the behavior of individual samples in simulated gastrointestinal fluids (Supplementary Figure S1e–h) revealed similar trends in the behavior of Rutile 5 in SGF, SGF^Pepsin^, and SIF, with an immediate increase in particle/agglomerate size from 287 ± 8 nm (in water) to ~500 nm after mixing with the respective fluid. With prolonged contact time, the particles gradually agglomerated, with maxima of around 1000 nm, which also corresponded to the behavior in PBS, DMEM, and DMEM^Serum^. In contrast, in SIF^Pancreatin^, the samples behaved differently, forming large agglomerations upon mutual contact. Similarly, for Anatase and Anatase/Rutile, particles agglomerated the most in SIF^Pancreatin^, with agglomeration decreasing in the following order: SGF^Pepsin^ < SIF < SGF. Conversely, the behavior of Rutile 1 differed considerably, which can be explained by the different particle morphology observed by SEM. In addition, the sizes of particles of Rutile 1, Anatase, and Anatase/Rutile remained almost constant in SGF throughout the measurement time.

### 3.4. Behaviour of TiO_2_ Particles in Human Blood Plasma

**Figure 7:**
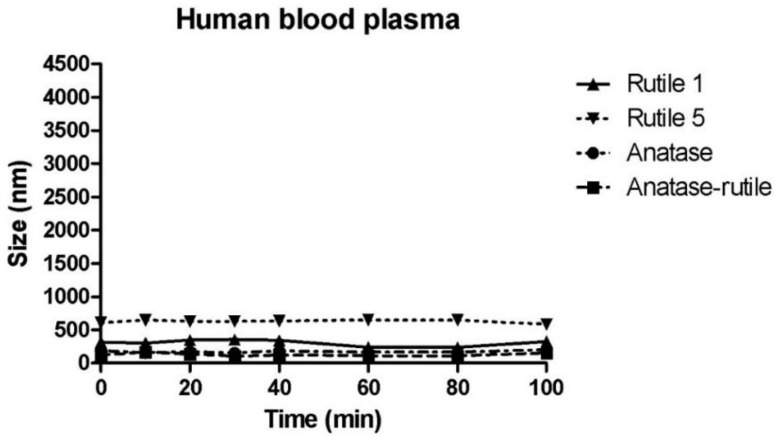
Time-dependent change in particle size of TiO_2_ particles in human blood plasma. The starting concentration of the TiO_2_ particles was 0.5 mg/mL dispersion media.

In contrast to the other tested fluids, all samples in blood plasma (Figure 7) changed less during the course of the measurement. The smallest change in particle size after direct mixing with blood plasma was observed with Rutile 1, with only a slight increase in size from 215 ± 1 nm (MilliQ water) to 318 ± 11 nm. Conversely, the particle size increased most markedly for Rutile 5 from the reference value of 287 ± 8 nm to the value of 607 ± 22 nm. In contrast, particles of Anatase/Rutile and Anatase samples were even smaller in plasma than in water. The low agglomeration of TiO_2_ particles in blood plasma compared to other fluids is conditioned by their rapid interactions with proteins the plasma contains, and the formation of a protein corona discussed above. Deng et al. studied the binding of human plasma proteins to commercially available metal oxides, including TiO_2_ NPs, and observed the agglomeration of TiO_2_ NPs in water, buffer, and biological media, which led to an increase in the amount and number of different proteins bound to these nanoparticles. The binding of plasma proteins to metal oxide NPs reached equilibrium during the first five minutes of incubation, which could explain the constant particle size observed throughout the measurement period in our study. Deng et al. further identified specific blood plasma proteins that bind to TiO_2_ NPs; namely, apolipoprotein A1, albumin, immunoglobulins (IgM and IgG), fibrinogen, and a number of other minor proteins [48]. Although albumin is the most plentiful protein in plasma, it was associated with metal oxides at fairly low levels. In a study conducted by Ruh et al., fibrinogen demonstrated the strongest affinity for all tested NPs, including TiO_2_, which could then hinder the adsorption of other proteins. In addition, the authors found that protein adsorption may depend on the crystal structure of TiO_2_ NPs, and reported, contrary to expectations, that smaller particles with a higher specific surface area do not bind more protein than larger particles [49]. Marucco et al. further found that both albumin and fibrinogen interact irreversibly with the negative surface of TiO_2_ NP where the interaction with albumin appears to be controlled by conformational changes of the protein at the surface due to its low resistance to deformation, while interactions with fibrinogen are conditioned by both conformational changes and electrostatic forces between TiO_2_ surface and the positive domains of the proteins [50]. According to Deng et al., the state of agglomeration greatly influences the binding of plasma proteins, and therefore, the resulting biodistribution may be, to a certain extent, dictated by the degree of agglomeration of nanoparticles, with this affecting cell surface protein binding [48].

### 3.5. Biological Properties

**Figure 8:**
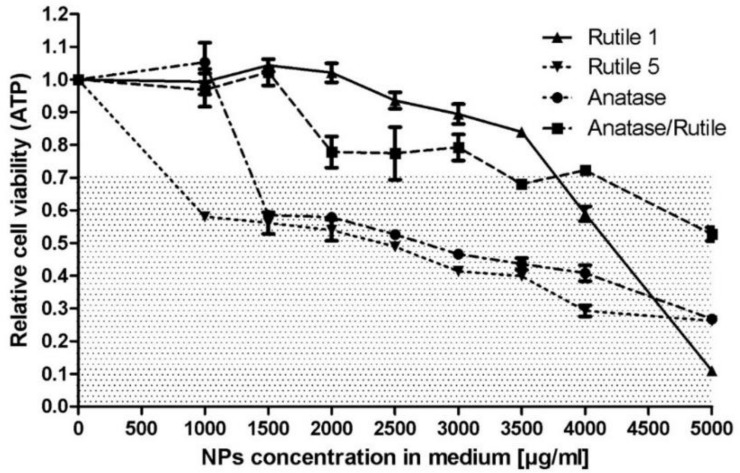
Cytotoxicity of TiO_2_ particles after 24 h of exposure to NIH/3T3 mouse embryonic fibroblast cells. The patterned area shows concentrations with cytotoxic effects, as defined by EN ISO 10993-5, where a viability > 0.7 corresponds to an absence of cytotoxicity.

Cytotoxicity is the principal and important test, which provides primary indication of particles behavior in contact with living systems. The aim of the experiment was to determine the toxic dose of TiO_2_ particles for NIH/3T3 cells. Concentrations for evaluating the cytotoxic effect were chosen following the study by Jin et al., who, similarly to our study, used mouse fibroblast cells (L929) for cytotoxicity testing. In their study, the cytotoxicity of weakly aggregated anatase TiO_2_ nanoparticles (in the concentration range of 3–600 µg/mL) was investigated, with the authors observing no significant reduction in viability after 24 h of cell culture [51]. Therefore, higher concentrations (1000–5000 μg/mL) were chosen for our study to determine doses with a significant impact on NIH/3T3 cells. The results of the experiment presented in Figure 8 show that cytotoxicity levels were different for each of the TiO_2_ samples. Rutile 1 was cytotoxic at a dispersion concentration of 4000 μg/mL, while Rutile 5 was cytotoxic at a dispersion concentration of 1500 μg/mL and, thus, more cytotoxic than Rutile 1. Anatase behaved similarly to Rutile 5, exhibiting a cytotoxic dose at a concentration of 1500 μg/mL. These differences may have arisen from the different physicochemical properties of the tested samples, whether these were the particle size or surface chemistry of the particles. 

Several studies on the cytotoxicity of commercial TiO_2_ NPs have been performed on different cell types but reported inconsistent results [52,53,54,55,56,57,58,59,60]. Differences in the cytotoxic effects of TiO_2_ NPs in these published studies may be the result of several factors such as the type of TiO_2_ NP, the type of cells used, the concentration of the tested range, etc. Some studies, similar to the one reported here, used mouse fibroblasts for the testing. Of these, Pittol et al., for example, observed no significant reduction in viability after the exposure of mouse fibroblast cells (L929) to TiO_2_ particles, even at the highest concentrations of 10,000 μg/g. The authors attributed the non-toxic effect of the particles to their agglomeration tendency, composition, and crystalline form, together with their interaction in culture media [61]. Similarly, Rosłon et al. observed only a slight effect of TiO_2_ NPs on the viability of mouse fibroblasts (L929)—however, at a notably lower range of tested concentrations (10–200 μg/mL) [62]. Again, similarly, Wagner et al. did not observe any toxic effect of TiO_2_ NPs on NIH/3T3 cells at a concentration of 0.1% (1000 μg/g) [63]. The concentrations of the particles tested in our study were relatively high and, therefore, the studied TiO_2_ samples should not pose a risk to human health.

Additional information on the fate of cells in contact with the studied particles was obtained by flow cytometry analysis, which proved that the cytotoxicity is mostly related to the necrosis and not to apoptosis of the cells (Supplementary Figure S2).

Simple verification of TiO_2_ particles dermal penetration of one Rutile and Anatase sample demonstrated absence of particles on the epidermal surface, which complies with literature findings (Supplementary Figure S3).

## 4. Conclusions

Titanium dioxide is widely used in various products, which necessitates a deep understanding of its impact on human health. This study presents a unique comparison of four different types of TiO_2_ particles with respect to their behavior in media used for biocompatibility testing, simulated fluids of the gastrointestinal tract, and plasma.

The results showed that all tested forms of TiO_2_ particles agglomerate immediately after contact with PBS and serum-free DMEM due to the presence of electrolytes that screen particle surface charge, with agglomeration levels increasing in the order: Rutile 5 < Anatase < Anatase/Rutile < Rutile 1. However, the presence of calf serum in DMEM significantly reduced agglomeration as a result of the formation of a protective protein corona around the particle surface. If we consider the general behavior of TiO_2_ in media routinely used for biological testing, the positive influence of serum in DMEM in reducing agglomeration in the medium is obvious. Thus, the in vitro cell culture studies dealing with biocompatibility of particles should take this finding into consideration when results are presented.

To mimic the digestive process, TiO_2_ particles were exposed to simulated saliva, stomach, and intestinal fluid, suitably supplemented with the digestive enzymes pepsin or pancreatin. The results demonstrate that, contrary to the protective action of calf serum in DMEM, pepsin and pancreatin (though being of a protein nature) trigger the significant agglomeration of TiO_2_ particles in SGF and SIF. The formation of an agglomerate was notably higher in comparison with the situation when the enzymes were absent. After mixing TiO_2_ particles with saliva, the samples behaved similarly as in PBS and DMEM with respect to the order of agglomeration level. When mixed with blood plasma, however, TiO_2_ agglomeration was significantly reduced thanks to the protective protein corona hindering interactions between TiO_2_ particles. In summary, the crystalline forms of TiO_2_ particles, ionic strength of surrounding media, and presence of proteins are all factors that influence colloidal stability of TiO_2_ particles in the media discussed above.

Cytotoxicity data determined with NIH/3T3 cells demonstrated an effect dependent on the type of tested TiO_2_ particles. The lowest cytotoxic effects were recorded for Rutile 1 and the Anatase/Rutile mixture, which were cytotoxic at TiO_2_ particle concentrations of 4000 and 3500 µg/mL, respectively. Higher cytotoxicity was yielded by Anatase and Rutile 5, which were cytotoxic at 1500 µg/mL.

## Figures and Tables

**Table 1:** Specification of TiO_2_ particles used in the study as given by suppliers.

Sample	Specific Surface Area [m^2^/g]	Crystalline Form	Crystal Size [nm]
Rutile 1	76.02	rutile	21
Rutile 5	7.11	rutile	150
Anatase	45–55	anatase	<25
Anatase/Rutile	n. p.	anatase/rutile	<100 ^a^, <50 ^b^

^a^ based on the specific surface area of samples as determined by BET; ^b^ based on X-ray diffraction.

**Table 2:** The diameter and PDI of TiO_2_ particles dispersed in water together with zeta potentials determined in water with pH adjusted to 7 and IEPs of studied samples.

Sample	Z-Average [nm]	PDI	Zeta Potential [mV]	IEP
Rutile 1	215 ± 1	0.23 ± 0.01	−21.6 ± 1.0	4.4
Rutile 5	287 ± 8	0.18 ± 0.01	−35.5 ± 0.9	4.0
Anatase	553 ± 7	0.33 ± 0.02	−18.5 ± 0.4	5.5
Anatase/Rutile	142 ± 2	0.35 ± 0.01	−3.0 ± 0.6	6.0

## Data Availability

The data presented in this study are available on request from the corresponding author.

## Supplementary Materials

The following are available online at https://www.mdpi.com/article/10.3390/ijms221910614/s1, Figure S1: The time-dependent change in particle size of TiO_2_ particles in media used for in vitro experiments (S1a–d), and in simulated body fluids (S1e–h), Figure S2: Flow cytometry analysis of the cell death type after fluorescent staining, Figure S3: Representative microscopic images of the strips and the *epidermis* of Anatase after 1st strip (A), 20th strips (C), and the *epidermis* surface after stripping (E), and Rutile 5 after 1st strip (B), 20th strips (D), and the *epidermis* surface after stripping (F).
