# Supplementary material for: Behaviour of Titanium Dioxide Particles in Artificial Body Fluids and Human Blood Plasma
Source: Int J Mol Sci. 2021 Sep 30;22(19):10614. doi: 10.3390/ijms221910614 (PMC8509028; doi:10.3390/ijms221910614)
Supplement: Supplementary file 1 [file ijms-22-10614-s001.zip › ijms-1377147-supplementary.pdf]

Supplementary material

Behaviour of titanium dioxide particles in artificial body fluids and human blood plasma

Eva Korábková et al.[a]

[a] Centre of Polymer Systems and Faculty of Technology, Tomas Bata University in Zlin, 760 01 Zlin, Czech Republic

Behaviour of TiO_2 particles in cultivation media

The experimental part describing the behaviour of TiO_2 particles in the tested fluids was expanded for better clarity by graphs summarizing the effect of the tested fluids on individual samples.

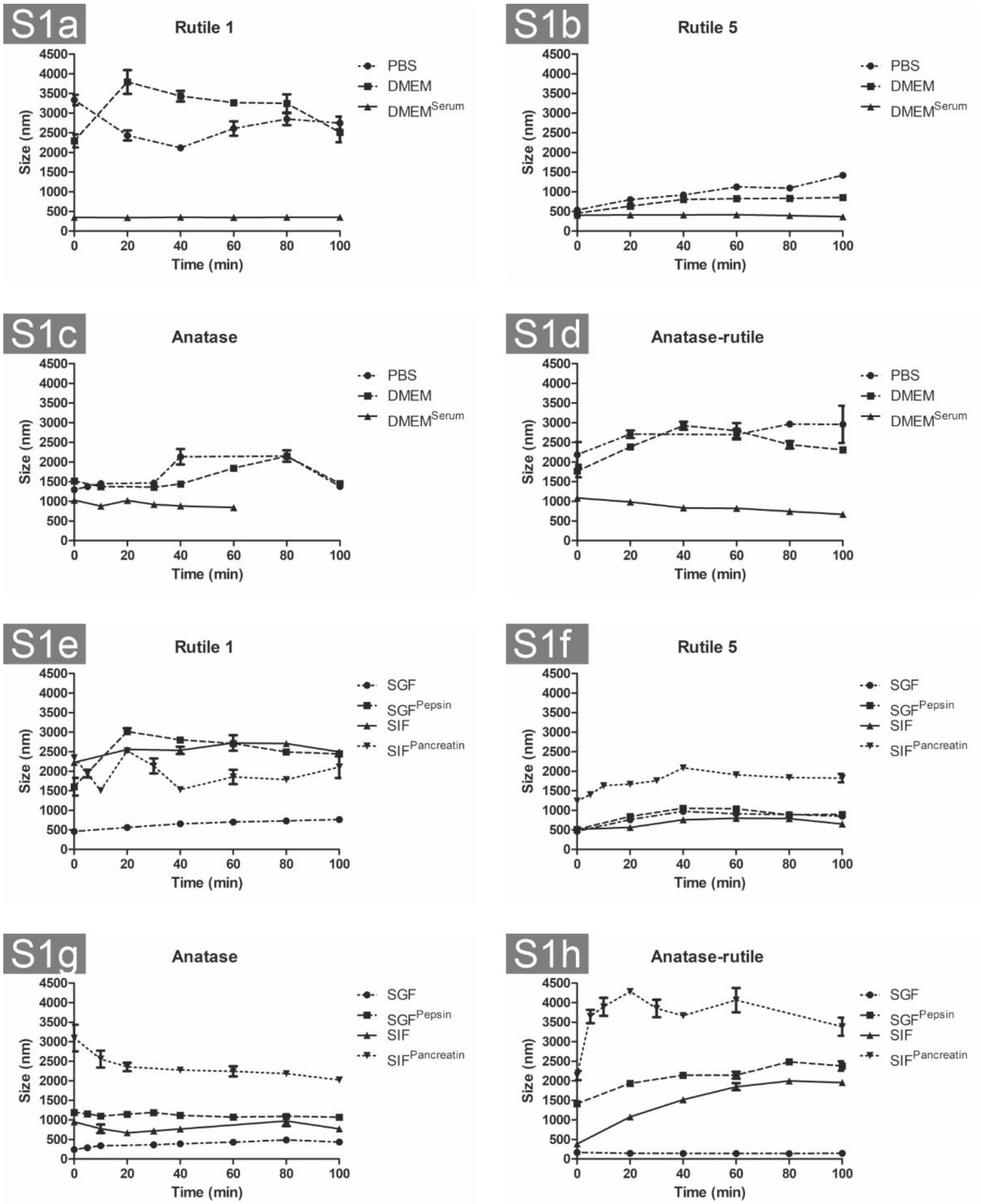

Figure S1. The time-dependent change in particle size of TiO_2 particles in media used for *in vitro* experiments (S1a-S1d), and in simulated body fluids (S1e-S1h).

Detection of cell death type

To distinguish the type of cell death (apoptosis or necrosis) staining with propidium iodide and Annexin V was employed.

Method: Firstly, fibroblasts cells were exposed to the TiO_2 dispersions in cytotoxicity test. The threshold concentrations determined in the cytotoxicity test were used in flowcytometry and the samples were tested in triplicates. Specifically, concentrations were as follow: rutile 1 4000 µg/ml, rutile 5 1000 µg/ml, anatase 1500 µg/ml, anatase/rutile 2000 µg/ml. After 24 hours of the cell exposure to the samples, the dispersions were removed, cells were washed with PBS and subsequently treated with trypsin. Detached cells were mixed with previously removed dispersons and stained by Annexin V-FITC (BD Biosciences, Canada) in a concentration of 2.5 g/ml and by propidium iodide (PI; BD Biosciences, Canada) in a concentration of 5 g/ml. After 15 minutes in the dark, cells were analysed on a BD FACSCanto flow cytometer (BD Biosciences, Canada).

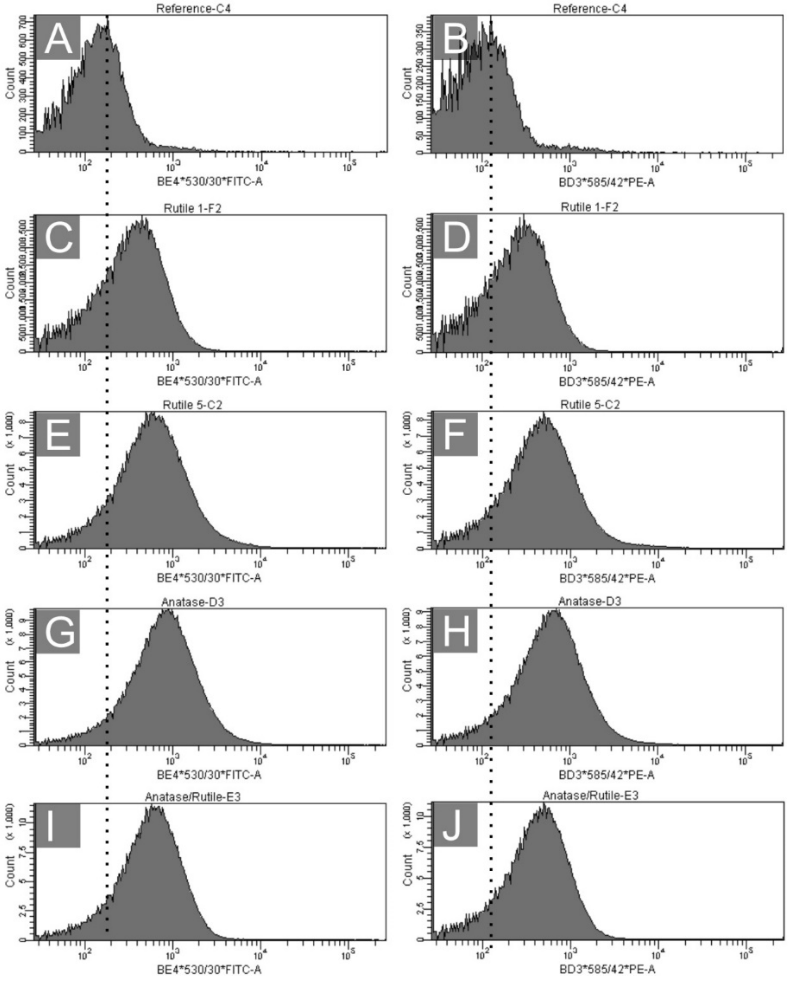

Figure S2. Flow cytometry analysis of the cell death type after fluorescent staining.

Results: Fluorescent staining with Annexin V-FITC and PI was used to detect apoptotic and necrotic cells population. Annexin V-positive/PI-negative cells indicate apoptotic and Annexin V-positive/PI-positive necrotic cells. As can be seen from figure S2, on cells treated with all the samples the fluorescent signal was substantially higher than on the reference (Fig. S2 A-B). That means that cells showed positivity for both the Annexin V and PI. The lowest signal was observed after treatment with Rutile 1 (Fig. S2 C-D), also the quantity of fluorescent positive cells was the lowest. The treatment with other samples (Fig. S2 E-J) has very similar effect on the type of the cell death as their fluorescent signal and width of the peaks was comparable. Based on the presented data, it can be concluded that observed cytotoxic effect is connected to the evoking of cell necrosis.

Transdermal absorption of TiO_2 particles

This experimental part investigated the penetration of TiO_2 particles after dermal exposure *in vitro*.

Methods: *In vitro* transdermal absorption of TiO_2 particles was determined according to OECD Test Guideline 428: Skin absorption: in vitro method with minor modifications using the porcine skin. The integrity of skin samples containing all layers of *epidermis* and *dermis* was determined by measuring transepidermal water loss (TEWL) (Tewameter® TM 300, Courage & Khazaka, Germany) before any experiment. Skin thickness was also measured using a micrometer (Kinex, Czech Republic). The samples of skin with a TEWL value not exceeding 15 $g/m^2/h$ were used. These values indicate good barrier function of the skin used (the Tewameter TM 300: Technical charges 2013). Skin samples with a similar thickness of around 1.65 ± 0.12 mm were selected for analysis.

One rutile and one anatase form of TiO_2 were selected for this experimental part. For analysis, 0.5 g of TiO_2 (Rutile 5 and Anatase) were sonicated in 10 mL caprylic/capric triglyceride (TCC) (Acetrade, Czech Republic) for 20 min using a UP400S sonicator (Heielscher, Germany). The transdermal diffusion detection system Permegear Fraction Collector FC33 (SES GmbH Analysesysteme, Germany) was used. The skin samples were inserted into the Franz chambers and left in contact with the receptor fluid for 30 minutes, before applying the test substance in final doses of tested samples, 10 $\mu l/cm^2$. The receptor fluid contained PBS, 0.05% gentamicin sulfate (Sigma Aldrich, Germany) and 1.5% surfactant (Brij® O20, Sigma Aldrich, Germany). After 24 h contact, transdermal absorption of TiO_2 particles was evaluated by stripping procedure. A total of 20 strips were obtained from each sample in duplicate. The strips were observed using an inverted Olympus phase contrast microscope (Olympus IX81, Japan).

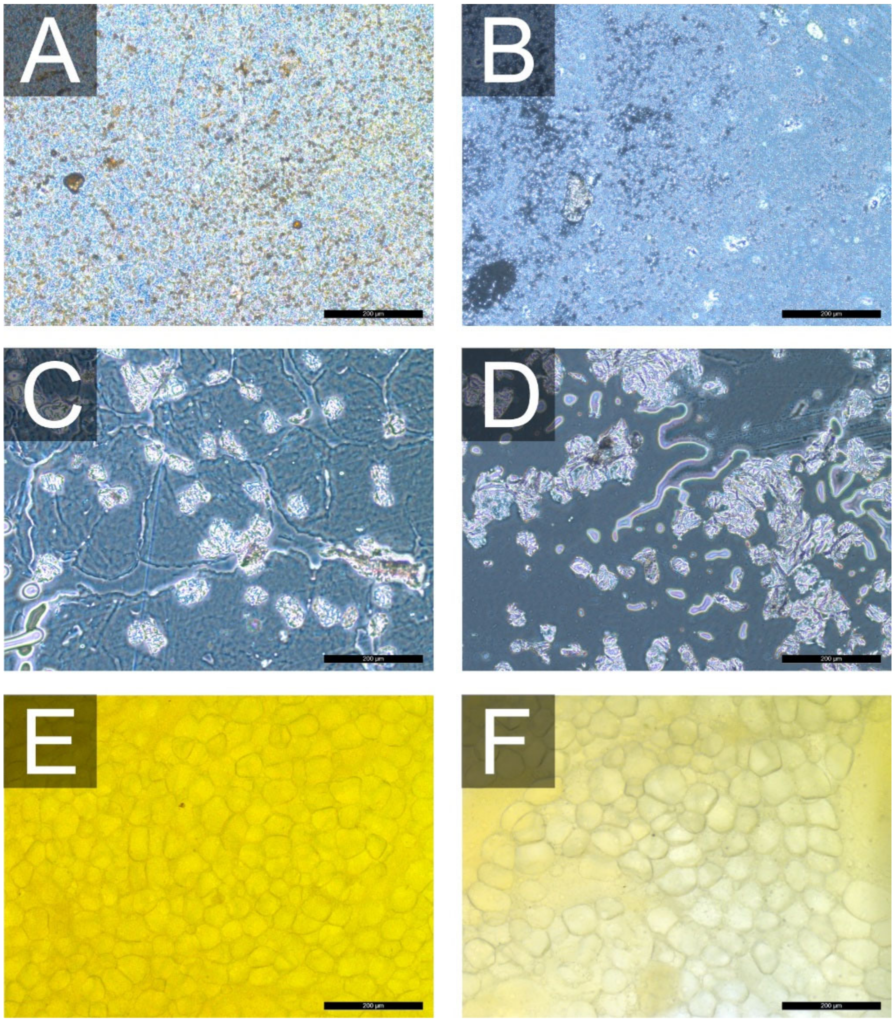

Figure S3. Representative microscopic images of the strips and the *epidermis* of Anatase after 1st strip (A), 20th strips (C), and the *epidermis* surface after stripping (E), and Rutile 5 after 1st strip (B), 20th strips (D), and the *epidermis* surface after stripping (F).

Results: Microscopic images (Fig. S3A-S3D) revealed the presence of TiO_2 particles (Anatase and Rutile 5) in the strips. Figure S3A and S3B represent the first strip from the sample and thus the highest concentration of particles. However, the particle concentration decreased with the number of strips performed (Fig. S3C and S3D). In addition, microscopy showed the absence of tested TiO_2 particles on the surface of the *epidermis* (Fig S3E and S3F), which is consistent with the literature data.
